# Quality Deterioration Kinetics and Arrhenius-Based Shelf-Life Prediction of Ready-to-Eat *Tremella fuciformis* Cold Dishes

**DOI:** 10.3390/foods15132260

**Published:** 2026-06-24

**Authors:** Rucai Chen, Wei Deng, Zhipeng Zheng, Yibin Li

**Affiliations:** 1 College of Tourism and Leisure Management, Fujian Business University, Fuzhou 350012, China; crccn950@126.com; 2 Institute of Food Science and Technology, Fujian Academy of Agricultural Sciences, Fuzhou 350003, China; ld199905@163.com (W.D.); zzp9701@163.com (Z.Z.); 3 College of Food Science, Fujian Agriculture and Forestry University, Fuzhou 350002, China; 4 Key Laboratory of Subtropical Characteristic Fruits, Vegetables and Edible Fungi Processing (Co-Construction by Ministry and Province), Ministry of Agriculture and Rural Affairs, Fuzhou 350003, China; 5 Fujian Province Key Laboratory of Agricultural Products (Food) Processing Technology, Fuzhou 350003, China

**Keywords:** edible fungi, ready-to-eat food, kinetic modeling, microbial spoilage, lipid oxidation, quality index

## Abstract

Shelf-life prediction is crucial for the industrial development and quality preservation of ready-to-eat (RTE) *Tremella fuciformis* cold dishes. In this study, we investigated the quality deterioration kinetics of *T. fuciformis* RTE dishes during storage at 5 °C, 15 °C, and 25 °C to establish an Arrhenius-based shelf-life prediction model. Throughout the storage period, the total bacterial count (TBC), viscosity, b* value, and malondialdehyde (MDA) content continuously increased, whereas firmness and sensory scores exhibited a negative correlation with storage time. The interpolated microbiological shelf-life endpoints, determined as the time required for TBC to reach 5.0 log CFU/g, were 19.01, 5.53, and 1.58 days at 5 °C, 15 °C, and 25 °C, respectively. Pearson correlation analysis identified TBC and MDA as the primary predictive indicators, with both parameters strictly conforming to zero-order reaction kinetics. Validation of the Arrhenius-based models revealed that the TBC-based model showed lower internal prediction error within the tested temperature range (average relative error of 8.92%), significantly outperforming the MDA model, which exhibited poor reliability (30.41% average relative error). These findings provide a practical reference for cold-chain management and shelf-life estimation of RTE *T. fuciformis* products for optimizing cold-chain management and ensuring the microbiological safety of RTE *T. fuciformis* products.

## 1. Introduction

*Tremella fuciformis* is a highly valued edible and medicinal mushroom renowned for its diverse physiological benefits, including antioxidant, hypolipidemic, and immunomodulatory properties [1,2]. As the leading global producer, consumer, and exporter, China dominates the *T. fuciformis* market, accounting for over 90% of worldwide production (approximately 536,000 tons of fresh weight) [3]. Traditionally, *T. fuciformis* is frequently consumed in China as a prepared cold dish, a culturally significant specialty food with considerable commercial potential. Despite its massive production scale and culinary popularity, the current industry relies heavily on primary processing, resulting in a distinct scarcity of value-added products. Therefore, the development of ready-to-eat (RTE) *T. fuciformis* cold dishes represents a promising strategy to deeply process this resource, diversify its utilization, and meet the growing consumer demand for convenient, health-promoting foods [4].

For the industrialization of RTE foods, accurate shelf-life prediction is essential for ensuring product safety. Traditional real-time shelf-life evaluations are often hindered by lengthy experimental periods and high costs [5]. With the advancement of interdisciplinary applications, an increasing number of mathematical models have been employed to predict the shelf life of food [6]. Currently, a widely adopted approach involves predicting shelf life based on chemical reaction kinetics while accounting for multiple quality deterioration factors [7]. Temperature is the most critical external variable influencing the chemical reaction rates in food; therefore, it serves as the benchmark for establishing dynamic shelf-life models [8]. The Arrhenius equation, a classical model for describing elementary chemical reactions, integrates various parameters and kinetic equations. It has been widely validated for predicting the shelf life of various foods, with its predictive performance depending on the specific food matrix, temperature range, kinetic indicator, and validation design [9].

*T. fuciformis* is characterized by its high moisture content (>90% after rehydration), near-neutral pH (approximately 6.3), and rich nutrient composition, making it highly susceptible to microbial spoilage. The addition of seasoning oils (rattan pepper oil and sesame oil, totaling approximately 20% of the formulation) introduces lipid substrates that are prone to oxidative rancidity during storage. Furthermore, while modified atmosphere packaging (MAP) with elevated CO_2_ levels can suppress aerobic microorganisms, it may selectively favor the growth of facultative anaerobic spoilage bacteria. These intrinsic and extrinsic factors collectively render RTE *T. fuciformis* cold dishes a microbiologically high-risk product that requires careful shelf-life management. Notably, *T. fuciformis* has been historically associated with food safety incidents involving bongkrekic acid, a potent toxin produced by *Burkholderia gladioli* pathovar *cocovenenans* (BGC). BGC growth and toxin production are favored by temperatures above 26 °C and neutral pH in high-moisture substrates. Therefore, verifying the absence of bongkrekic acid within the established shelf life is a critical safety verification for any *T. fuciformis*-based RTE product.

Despite the proven efficacy of these kinetic models in the beef [10] and dairy sectors [11], comprehensive modeling for high-moisture, mushroom-based RTE foods is severely lacking. While previous research has primarily focused on optimizing the formulation and packaging of RTE *T. fuciformis* cold dishes, the dynamic quality changes and actual shelf lives of these products under varying storage temperatures remain poorly understood. Therefore, this study aims to systematically investigate the physical, chemical, and microbiological deterioration kinetics of these RTE dishes during storage at 5 °C, 15 °C, and 25 °C. By identifying key quality indicators that are highly correlated with product spoilage, we seek to construct and validate a reliable shelf-life prediction model utilizing the Arrhenius equation. Ultimately, this research provides a vital theoretical framework for optimizing preservation technologies, guiding cold-chain management, and ensuring strict quality control during the commercialization of RTE *T. fuciformis* products.

## 2. Materials and Methods

### 2.1. Materials and Reagents

Dried *T. fuciformis* (Tr 2016, moisture content 12.5% ± 1.2% *w*/*w*) was provided by Fujian Xiangyun Biotechnology Development Co., Ltd. (Fuzhou, China). The dried fruiting bodies had a yellowish-white color with characteristic lobed and folded morphology (8–15 cm diameter when dried). The microbiological quality of the raw dried *T. fuciformis* was: aerobic plate count < 10^3^ CFU/g, coliforms < 10 CFU/g, molds and yeasts < 10^2^ CFU/g.

Rattan pepper oil (food-grade, peroxide value < 5.0 meq/kg, acid value < 3.0 mg KOH/g) was sourced from Sichuan Hongya County Yajiang Food Co., Ltd. (Emeishan, China). Sesame oil (food-grade, peroxide value < 4.0 meq/kg, acid value < 2.0 mg KOH/g) was sourced from COFCO Group Co., Ltd. (Beijing, China). Edible salt (China National Salt Industry Corporation., Beijing, China) and monosodium glutamate (Fufeng Group Co., Ltd., Qingdao, China) were food-grade. All other chemicals and solvents were of analytical grade and purchased from Sinopharm Chemical Reagent Co., Ltd. (Shanghai, China).

### 2.2. Preparation and Storage of RTE T. fuciformis Cold Dishes

Rehydration: Dried *T. fuciformis* was rehydrated using a KQ-500DE ultrasonic cleaner (Kunshan Ultrasonic Instruments Co., Ltd., Suzhou, China; power: 500 W; frequency: 40 kHz) in 100 °C deionized water for 3 min. The water-to-sample ratio was 10:1 (*w*/*w*). After rehydration, the samples were drained and cooled to room temperature (approximately 25 °C) on a stainless-steel mesh for 15 min. The final moisture content after rehydration was 92% (*w*/*w*). Dried and rehydrated *T. fuciformis* are shown in Figure S1.

Formulation: For every 100 g of rehydrated *T. fuciformis*, the following seasoning formulation (previously optimized by our group using response surface methodology for consumer acceptability) was added: 9.66% rattan pepper oil, 1.94% edible salt, 1.56% monosodium glutamate, and 10% sesame oil. All ingredients were mixed thoroughly for 2 min to ensure uniform distribution.

Packaging: The prepared samples (approximately 100 g per package) were packaged in polyethylene (PE) composite film bags (thickness: 80 μm; oxygen transmission rate < 50 cm^3^/(m^2^·24 h·0.1 MPa) at 23 °C and 50% RH; water vapor transmission rate < 5 g/(m^2^·24 h) at 38 °C and 90% RH). The packages were flushed with a modified atmosphere consisting of 40% CO_2_ and 60% N_2_ using a DZ-400/2S vacuum packaging machine (Zhejiang Dongfeng Packaging Machinery Co., Ltd., Wenzhou, China) with gas flushing function, at a gas-to-product volume ratio of approximately 2:1, and then heat-sealed. Residual oxygen in the headspace was verified to be <1% using a PBI Dansensor CheckMate 3 gas analyzer (AMETEK MOCON, Ringsted, Denmark).

Storage Conditions: The packaged RTE cold *T. fuciformis* samples were stored in artificial climate incubators (MGC-450HP, Shanghai Yiheng Scientific Instruments Co., Ltd., Shanghai, China) set at 5 °C, 15 °C, and 25 °C, representing refrigerated storage, mild temperature abuse, and ambient/accelerated conditions, respectively. These temperatures were selected to cover the practical range encountered during cold-chain distribution and to provide sufficient spacing for Arrhenius modeling. Sampling was conducted every 2 days (Days 2 to 22) for the 5 °C group; every 1 day (Days 1 to 6) for the 15 °C group; and every 0.5 days (Days 0.5 to 1.5) for the 25 °C group. Sampling frequencies were determined based on preliminary experiments. A total of 63 packages were prepared (33 for 5 °C, 18 for 15 °C, and 9 for 25 °C, plus 3 for initial analysis), with three independent packages analyzed per temperature per time point (destructive sampling; each package was opened only once).

### 2.3. Color Measurement

The color parameters of the rehydrated *T. fuciformis* were measured using an NS810 spectrophotometer (3nh Co., Shenzhen, China) with a D65 illuminant and 10° observer angle. The instrument was calibrated against a standard white calibration plate (L* = 93.80, a* = −0.32, b* = 0.56) before each measurement session. Five pieces of *T. fuciformis* fruiting body were randomly selected from each package, and the flat central region of each piece was measured to record L* (lightness), a* (redness/greenness), and b* (yellowness/blueness) values. Three independent packages were measured per time point (15 readings total). Total color difference (ΔE) was calculated as ΔE = [(ΔL*)^2^ + (Δa*)^2^ + (Δb*)^2^]½, and Browning Index (BI) was calculated using the formula BI = [100 × (x − 0.31)]/0.17, where x = (a* + 1.75L*)/(5.645L* + a* − 3.012b*).

### 2.4. Texture Measurement

The smooth and intact fruiting body pieces of *T. fuciformis* were placed on the texture analyzer for measurement. The texture profile analysis (TPA) parameters (TA-XT22 Model, Haslemere, England) were set as follows: trigger mode, auto; pre-test speed, 1 mm/s; test speed, 2 mm/s; post-test speed, 2 mm/s; trigger force, 25 g; and compression ratio, 50%. Firmness and viscosity were derived from the built-in Tremella program of the texture analyzer.

### 2.5. Determination of Bongkrekic Acid Content

The bongkrekic acid content was determined with slight modifications to the protocol described by Wang [12], using high-performance liquid chromatography (HPLC) (Waters2695, Milford, MA, USA) with a quaternary pump and diode array detector (DAD). Chromatographic separation was performed on an Agilent ZORBAX Eclipse Plus C18 column (4.6 mm × 250 mm, 5 μm particle size) maintained at 30 °C. The mobile phase was methanol: water (75:25, *v*/*v*, containing 0.1% formic acid) at an isocratic flow rate of 1.0 mL/min. The injection volume was 20 μL, and detection was performed at 267 nm. A bongkrekic acid standard (purity ≥ 98%, Sigma-Aldrich, St. Louis, MO, USA) was used for calibration over the range 0.1–50 μg/mL (R^2^ = 0.9996). The limit of detection (LOD) was 0.03 μg/mL (S/N = 3), and the limit of quantification (LOQ) was 0.1 μg/mL (S/N = 10). Recovery rates ranged from 92.3% to 104.7% at three spiked levels (1, 5, and 10 μg/g), with intra-day RSD < 3.5% and inter-day RSD < 5.2%. Matrix-matched calibration standards were prepared using blank *T. fuciformis* matrix extract to compensate for potential matrix effects. All *T. fuciformis* samples were first thoroughly homogenized to disrupt the tissue structure and facilitate toxin release. The resulting homogenate was centrifuged at 8000 rpm for 10 min. The supernatant was then carefully collected and stored at 4 °C to serve as the crude extract. Afterwards, a 0.3 mL aliquot of this crude extract was mixed with 1 mL of methanol, vortexed thoroughly, and centrifuged at 2000 rpm for 3 min. The final clear supernatant was subjected to HPLC for the determination of bongkrekic acid.

### 2.6. Determination of Total Bacterial Count (TBC)

TBC was evaluated following the “GB 4789.2-2022 National Food Safety Standard—Food Microbiological Examination: Aerobic Plate Count.” [13]. A 25 g sample of RTE *T. fuciformis* cold dish was weighed under sterile conditions and homogenized in 225 mL of 0.85% (*w*/*v*) sterile saline for 2 min using a BagMixer 400 stomacher (Interscience Co., St Nom, France). A tenfold dilution series (10^−1^ to 10^−7^) was prepared using the same saline solution. Subsequently, 1 mL aliquots of three appropriate dilutions were inoculated onto plate count agar (PCA) in duplicate plates and incubated at 36 ± 1 °C for 48 ± 2 h. Three independent packages were analyzed per time point per temperature (destructive sampling). Results were expressed as log CFU/g. 

### 2.7. Determination of Malondialdehyde (MDA) Content

The MDA content was quantified using the thiobarbituric acid (TBA) method, with slight modifications to the protocol described by Sun [14]. The method was validated for the oil-rich *T. fuciformis* matrix through spike-and-recovery experiments using 1,1,3,3-tetraethoxypropane (TEP, purity ≥ 97%, Sigma-Aldrich) as the standard at concentrations of 0–10 nmol/mL (R^2^ = 0.9993). Recovery rates at three spiking levels (0.5, 2.0, and 5.0 nmol/g) ranged from 88.6% to 102.3%, with RSD < 6.8%. MDA content was expressed on a fresh weight basis (nmol/g).

Briefly, 5.0 g of the sample was homogenized with 25.0 mL of 10% trichloroacetic acid (TCA) solution in an ice-water bath. The mixture was then centrifuged at 10,000 rpm and 4 °C for 20 min. Subsequently, 2.0 mL of the supernatant was collected, mixed with 2.0 mL of 0.67% TBA solution, and heated in a boiling water bath for 20 min. After cooling to room temperature, the absorbance was sequentially measured at 600 nm, 532 nm, and 450 nm using a TU-1810 UV-Vis spectrophotometer (Beijing Purkinje GENERAL Instrument Co., Ltd., Beijing, China). For the blank control, the sample supernatant was replaced with 2.0 mL of 10% TCA solution. The MDA concentration was calculated using Equations (1) and (2). To minimize interference from co-extracted TBA-reactive substances (TBARS) such as alkenals and alkadienals from rattan pepper oil and sesame oil, the TCA extraction method was used rather than direct heating of the sample, and correction for non-MDA TBARS was performed by measuring absorbance at three wavelengths.

(1) $$MDA\left(\mathrm{nmol}\cdot g^{-1}\right)=\frac{c\times v}{v_{s}\times m\times 1000}$$

(2) $$c\left(\mathrm{nmol}\cdot g^{-1}\right)=\left[6.45\times \left(OD_{532}-OD_{600}\right)-0.56\times OD_{450}\right]$$

Here, *c* represents the MDA concentration in the supernatant (nmol·g^−1^); *v* is the total volume of the extract (mL); *v_s_* is the volume of the supernatant used for the reaction (mL); and m is the sample mass (g).

### 2.8. Sensory Evaluation

A sensory evaluation panel consisting of 10 trained members (5 males and 5 females, aged 22–35 years) was established from staff and students of the Institute of Food Science and Technology. Panelists were trained over three sessions (2 h each) within one week: Session 1 involved familiarization with sensory attributes, descriptors, and the scoring scale using freshly prepared and intentionally aged samples; Sessions 2 and 3 involved practices for scoring under conditions identical to the actual evaluation. Panelist performance was validated using the panel check method, requiring a coefficient of variation < 20% for each attribute. Evaluations were conducted in individual booths under white fluorescent lighting (ISO 8589:2007) at the Sensory Evaluation Laboratory [15].

The detailed scoring criteria are provided in Table S1. All samples were presented on white plates and assigned randomized three-digit codes. Samples were served at room temperature (22–25 °C). Panelists rinsed their mouths with purified room-temperature water between tastings to prevent flavor carryover. For samples approaching or exceeding the TBC threshold (5.0 log CFU/g), sensory evaluation was conducted only after confirming the absence of bongkrekic acid (<1 μg/kg). Panelists were instructed to expectorate all samples rather than swallow them. The sensory evaluation protocol was reviewed and granted exemption from formal ethics committee approval by the Institutional Review Board of Fujian Academy of Agricultural Sciences, as it involved only taste, smell, and texture assessment of safe food samples without the collection of personal and sensitive information.

### 2.9. Establishment of Shelf-Life Prediction Model

The shelf-life prediction models were developed by adapting the method described by Zhang [16]. TBC data (in log CFU/g) and MDA data (in nmol/g) for the three temperature groups were fitted with zero-order (*A* = *A*_0_ + *kt*) and first-order (*A* = *A*_0_ × *e^kt^*) kinetic models. For TBC, *A*_0_ (initial TBC) = 1.48 log CFU/g and *B* (critical limit) = 5.0 log CFU/g. For MDA, *B*_0_ (initial MDA) = 0.18 nmol/g and *B* (critical endpoint) = 0.60 nmol/g, defined as the MDA concentration at the TBC-determined microbiological shelf-life endpoint. Model selection was based on the coefficient of determination (R^2^). The Arrhenius equation (*lnk* = *lnk*_0_ − *Ea*/*RT*) was then used to establish the temperature dependence of the reaction rate constant (*k*) through linear regression of ln *k* against 1/*T*. The final shelf-life (*SL*) prediction model was derived by integrating the selected kinetic model with the following Arrhenius equation: *SL* = (*B* − *B*_0_)/[*k*_0_ × exp(−*Ea*/*RT*)].

Zero-order reaction kinetics:

$$A=A_{0}+kt$$

First-order kinetic model:

$$A=A_{0}\times e^{kt}$$

Arrhenius equation:

$$lnk=lnk_{0}-\frac{E_{a}}{RT}$$

where *A* represented the parameter value at time t, *A*_0_ represented the initial value, *k* represented the reaction rate constant (d^−1^), *t* represented storage time (d), *k*_0_ represented the pre-exponential factor, *Ea* represented activation energy (kJ·mol^−1^), *T* represented absolute storage temperature (K), and *R* represented the gas constant [8.314 J·(mol·K)^−1^].

### 2.10. Data Processing and Statistical Analysis

All experiments were performed using three independent packages per temperature per time point (biological replicates). Separate packages were used at each time point (destructive sampling design). Data are expressed as the mean ± standard deviation (SD). All figures were generated using OriginPro 2018 (OriginLab Corp., Northampton, MA, USA).

## 3. Results

### 3.1. Effects of Storage Temperature on TBC

**Figure 1:**
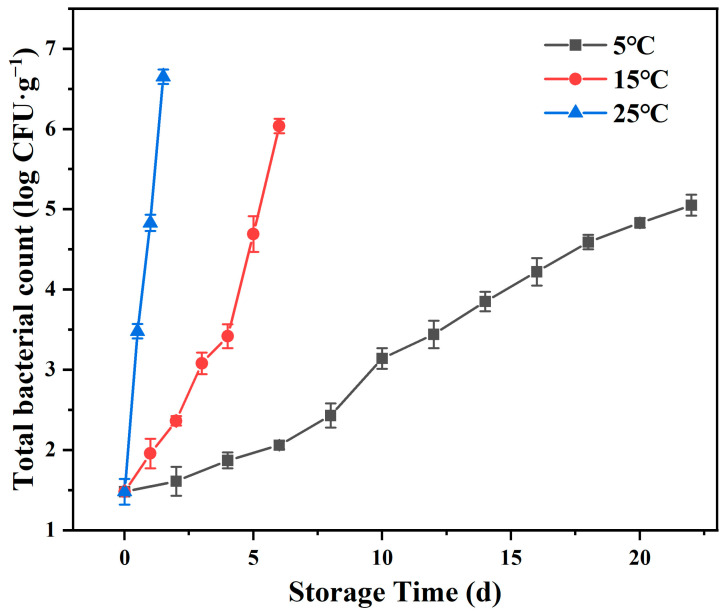
The total bacterial count of RTE *T. fuciformis* cold dishes under different storage temperatures during storage.

The maximum acceptable TBC limit for RTE cold dishes was set at 1 × 10^5^ CFU/g (5.0 log CFU/g) in accordance with the published literature [17,18,19]. Storage temperature exerted a profound effect on the TBC of the RTE *T. fuciformis* cold dishes, with higher temperatures substantially accelerating microbial proliferation (Figure 1). The microbiological shelf-life endpoints, determined by interpolation to exactly 5.0 log CFU/g using the zero-order kinetic equation, were 19.01, 5.53, and 1.58 days at 5 °C, 15 °C, and 25 °C, respectively. At 5 °C, TBC increased steadily from an initial value of 1.48 ± 0.15 log CFU/g to 5.0 log CFU/g at 19.01 days. At 15 °C, the same threshold was reached at 5.53 days, and at 25 °C, the most rapid growth was observed, with TBC reaching 5.0 log CFU/g within 1.58 days. Throughout the storage period, the TBC of the 5 °C group exhibited a steady linear increase (R^2^ = 0.986), whereas the 25 °C group displayed a more rapid but still approximately linear accumulation pattern (R^2^ = 0.941).

### 3.2. Effects of Storage Temperature on Color Parameters

**Figure 2:**
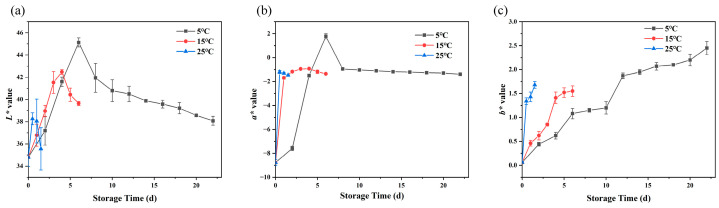
The L* value (**a**), a* value (**b**), and b* value (**c**) of RTE *T. fuciformis* cold dishes under different storage temperatures during storage.

**Figure 3:**
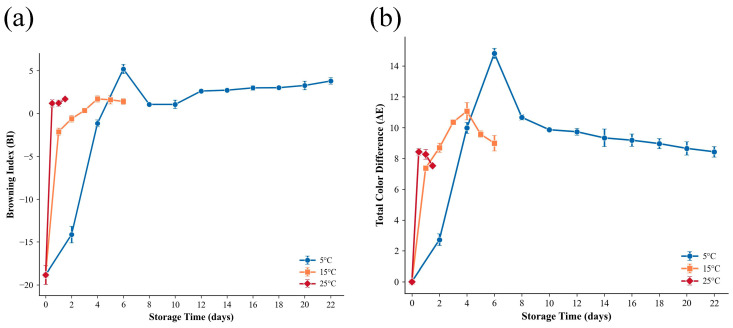
The BI (**a**) and ΔE (**b**) of RTE *T. fuciformis* cold dishes at different storage temperatures over a storage period.

The color parameters (L*, a*, and b* values) of the RTE cold dishes underwent continuous alterations throughout the storage period across all temperature groups. Regarding lightness (L*), the 5 °C group reached a maximum value of 45.14 ± 0.41 before exhibiting a gradual decline (Figure 2a). The maximum L* value for the 15 °C group was 42.48 ± 0.21. In contrast, the 25 °C group maintained consistently lower L* levels throughout the experiment, rapidly dropping to 35.55 ± 1.09 at its endpoint. For the redness/greenness coordinate (a*), the 5 °C group peaked at 1.78 ± 0.21, followed by a plateau and a subsequent slight decline during the later stages of storage (Figure 2b). The a* values for the 15 °C and 25 °C groups remained generally lower, with the 25 °C group rapidly decreasing to a minimum of −1.20 ± 0.13 by Day 0.5. Concurrently, the yellowness (b* value) exhibited a continuous upward trend across all temperatures (Figure 2c). The 15 °C and 25 °C groups showed marked increases, reaching 1.55 ± 0.11 and 1.68 ± 0.07 at their respective endpoints, whereas the 5 °C group reached a final maximum of 2.45 ± 0.14. The BI trajectories reveal a pronounced temperature dependency consistent with Arrhenius-type kinetics governing both enzymatic and non-enzymatic browning reactions. At 5 °C, BI progressed gradually from −18.8 to +3.8 over 22 days, whereas at 25 °C, BI crossed zero within 12 h and reached +1.7 within just 1.5 days. This is a value characteristic of polyphenol oxidase (PPO)-catalyzed reactions in edible fungi (Figure 3a). A striking and unexpected observation is the non-monotonic trajectory of ΔE across all temperatures. Rather than increasing monotonically, ΔE exhibited a distinct peak followed by a partial decline. At 5 °C, ΔE rose sharply from 0 to 14.8 by Day 6, then gradually decreased to 8.4 by Day 22. At 15 °C, ΔE peaked at 11.1 on Day 4 before declining to 9.0 by Day 6. Even at 25 °C, ΔE decreased from 8.4 at Day 0.5 to 7.5 at Day 1.5 (Figure 3b).

### 3.3. Effects of Storage Temperature on Texture

**Figure 4:**
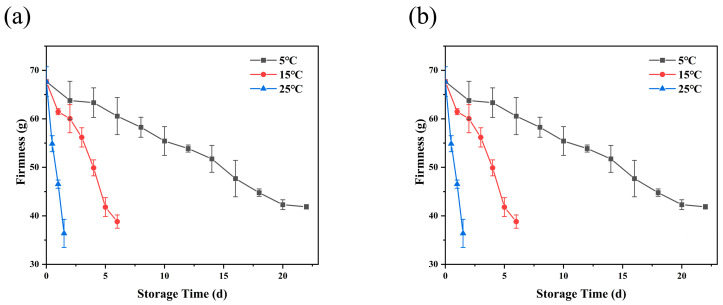
The firmness (**a**) and viscosity (**b**) of RTE *T. fuciformis* cold dishes under different storage temperatures during storage.

Elevated storage temperatures significantly accelerated the textural degradation of the RTE cold dishes, characterized by a continuous decline in firmness (Figure 4a) and a concurrent rise in viscosity (Figure 4b) over time. In the 5 °C group, firmness gradually decreased to 41.87 ± 0.46 g, while viscosity increased to 0.42 ± 0.03 g·s by the storage endpoint at Day 22. In the 15 °C group, firmness dropped to 38.81 ± 1.37 g, with viscosity rising to 0.45 ± 0.02 g·s by Day 6. Notably, the 25 °C group experienced the most rapid textural deterioration, with firmness plummeting to 36.39 ± 2.90 g and viscosity increasing to 0.43 ± 0.02 g·s within a mere 1.5 days.

### 3.4. Effects of Storage Temperature on Bongkrekic Acid Content

Bongkrekic acid remained undetectable (<1 μg/kg) in all samples across the three storage temperatures throughout their respective storage periods. This result confirms that the interpolated microbiological shelf-life endpoints (19.01 d at 5 °C, 5.53 d at 15 °C, and 1.58 d at 25 °C) effectively prevented the formation of bongkrekic acid within the effective shelf life of the products.

### 3.5. Effects of Storage Temperature on MDA Content

**Figure 5:**
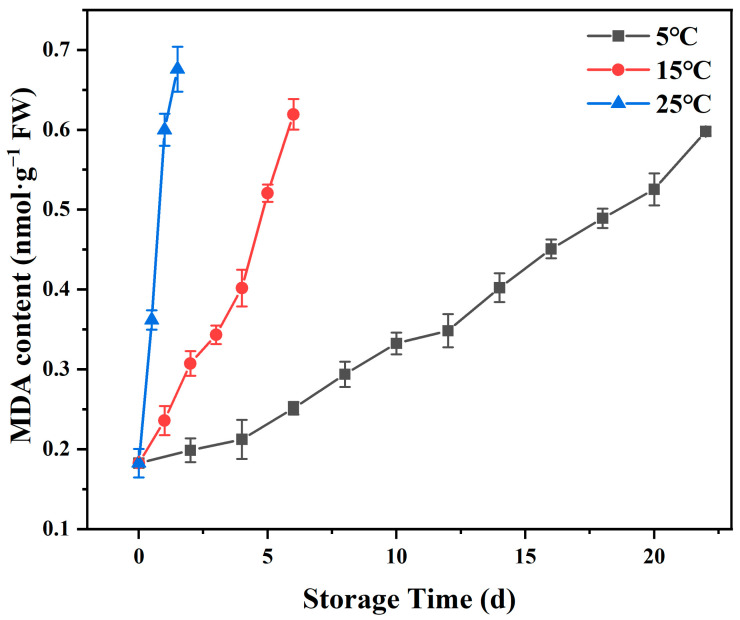
The MDA content of RTE *T. fuciformis* cold dishes under different storage temperatures during storage.

The MDA content, a primary biomarker of lipid oxidation, exhibited a continuous, temperature-dependent accumulation during storage. From an initial MDA content of 0.18 ± 0.02 nmol·g^−1^, higher storage temperatures markedly accelerated the rate of MDA generation (Figure 5). By the end of their respective storage periods, the final MDA contents for the 5 °C, 15 °C, and 25 °C groups escalated to 0.60 ± 0.01 nmol·g^−1^, 0.62 ± 0.02 nmol·g^−1^, and 0.68 ± 0.03 nmol·g^−1^, respectively.

### 3.6. Effects of Storage Temperature on Sensory Scores

**Figure 6:**
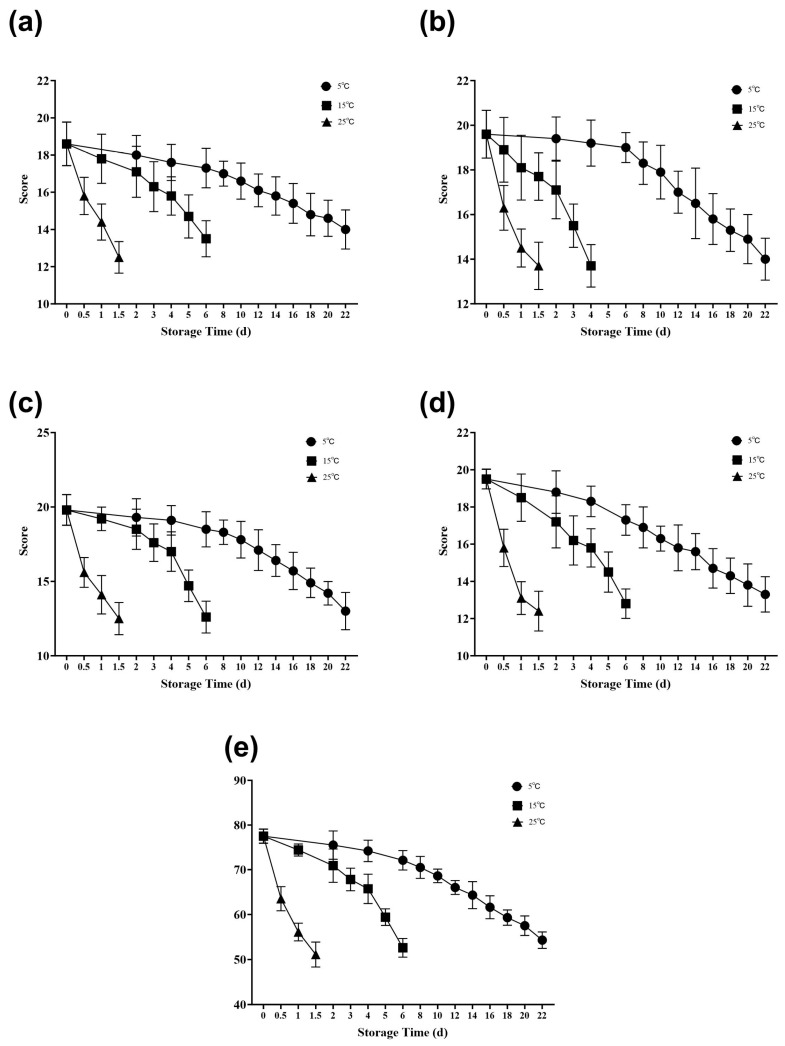
Sensory score of RTE *T. fuciformis* cold dishes by color (**a**), taste (**b**), flavor (**c**), taste (**d**), and total score (**e**) under different storage temperatures during storage.

The sensory scores, encompassing color, aroma, texture, and taste, deteriorated progressively as storage time increased across all temperature groups. Higher storage temperatures triggered drastically sharper declines in all sensory attributes (Figure 6). At 25 °C, severe quality loss was observed by the final sampling point, which was close to the interpolated microbiological shelf-life endpoint of 1.58 d., with scores dropping to 12.50 ± 1.85, 12.50 ± 1.08, 12.40 ± 1.07, and 13.70 ± 1.06 for color, aroma, texture, and taste, respectively. In terms of overall sensory evaluation, the final scores for the 5 °C, 15 °C, and 25 °C groups decreased by 29.94%, 32.10%, and 34.10% from their initial baseline values, respectively.

### 3.7. Correlation Coefficient Analysis of Physicochemical Indicators

Pearson correlation analysis revealed highly significant relationships among physicochemical and microbiological indicators during storage (Table 1). While the color parameters (L*, a*, and b*) displayed relatively lower correlations with other metrics, the absolute correlation coefficients among firmness, viscosity, TBC, and MDA content all exceeded 0.95. Notably, the correlation coefficient between TBC and MDA content was greater than 0.97, demonstrating an extremely significant positive correlation (*p* < 0.01). Based on these strong statistical relationships, TBC and MDA were selected as the primary indicators for subsequent shelf-life modeling.

### 3.8. Determination of the Shelf-Life Prediction Model

#### 3.8.1. Determination of Reaction Order

The degradation kinetics of both TBC and MDA were more accurately described by the zero-order reaction model than the first-order model. As detailed in Table 2, the determination coefficients (R^2^) for the zero-order kinetics of both indicators exceeded 0.95 across all temperatures. For TBC, the sum of R^2^ (∑R^2^) for the zero-order reaction was 2.9413, which was higher than that of the first-order reaction (2.8960). Similarly, for MDA, the zero-order ∑R^2^ (2.9328) surpassed the first-order ∑R^2^ (2.9035). Consequently, zero-order reaction kinetics were selected to model these indicators.

#### 3.8.2. Establishment of the Arrhenius Equation

**Figure 7:**
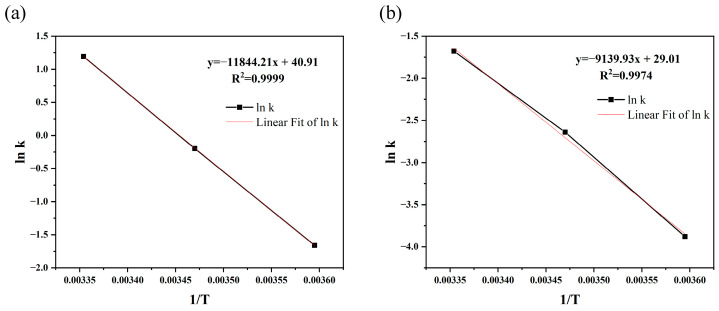
The linear (TBC (**a**) and MDA (**b**)) relationship between 1/T and ln *k*.

Arrhenius equations were successfully established by performing linear regression of ln *k* against 1/T. For TBC (Figure 7a), the fitted equation was y = −11,844.21x + 40.91 (R^2^ = 0.9999), yielding an activation energy (*Ea*) of 98,472.76 J·mol^−1^ and a pre-exponential factor (*k*_0_) of 5.86 × 10^17^. For MDA (Figure 7b), the equation was y = −9139.93x + 29.01 (R^2^ = 0.9974), resulting in *Ea* = 75,989.38 J·mol^−1^ and *k*_0_ = 3.97 × 10^12^. The final zero-order Arrhenius shelf-life prediction models based on TBC (Equation (3)) and MDA (Equation (4)) were derived accordingly.

(3) $$SL=\frac{B-B_{0}}{5.86\times 10^{17}\times e^{\frac{-11844.2}{T}}}$$

(4) $$SL=\frac{B-B_{0}}{3.97\times 10^{12}\times e^{\frac{-9139.93}{T}}}$$

#### 3.8.3. Verification of the Shelf-Life Model

Model validation revealed a stark contrast in predictive accuracy between the two indicators (Table 3). The MDA-based shelf-life prediction model exhibited poor reliability, with an average relative error of 30.41% and a maximum error of 70.67% at 25 °C. Conversely, the TBC-based model showed lower internal prediction error than the MDA-based model within the tested temperature range. Notably, the relative error for the TBC model at 25 °C was a mere 5.33%, indicating acceptable internal agreement for estimating shelf life under the tested storage temperatures.

## 4. Discussion

Temperature is a critical thermodynamic factor that dictates microbial metabolism and enzyme efficiency during food storage [20]. In this study, the rapid exponential increase in the TBC of RTE cold *T*. *fuciformis* dishes at 25 °C can be primarily attributed to the synergistic effect of a high-moisture matrix and elevated ambient temperatures. Such permissive environments provide optimal conditions for microbial enzymatic reactions, thereby drastically accelerating cellular metabolism and protein synthesis. Conversely, cold storage at 5 °C effectively suppressed microbial proliferation. This inhibitory effect aligns with the findings of Li, who reported a significant reduction in the TBC growth rate of postharvest shiitake mushrooms stored at 5 °C [21]. Similarly, Kim demonstrated that refrigeration effectively mitigated both TBC and coliform growth in ready-to-use salted napa cabbage, further corroborating the efficacy of low-temperature control in preserving high-moisture, plant-based RTE foods [22]. In the present study, bongkrekic acid generated by *Burkholderia gladioli* pathovar *cocovenenans* (BGC) was not detected in the samples. Several studies have consistently shown that the optimal temperature range for BGC growth is between 30 and 37 °C, and its toxin production is nearly completely inhibited at temperatures below 15 °C [23,24]. Furthermore, Yao indicated that substantial bongkrekic acid production typically initiates only after 4 days of incubation at 26 °C [25]. Because the actual shelf life of the RTE cold dishes at 25 °C was limited to 1.5 days due to the TBC safety threshold, the product spoiled before the pathogenic conditions required for bongkrekic acid accumulation were met. These findings provide robust evidence that the current processing for these RTE dishes inherently mitigates the risk of BGC poisoning.

The sensory deterioration of RTE cold *T. fuciformis* dishes, characterized by tissue softening, severe browning, and off-odor generation, is a complex process driven by synergistic enzymatic activities and lipid oxidation. Under low-temperature conditions (5 °C), the generation and mobility of reactive oxygen species (such as superoxide radicals, hydrogen peroxide, and hydroxyl radicals) are largely suppressed. This suppression mitigates membrane lipid peroxidation, delays MDA accumulation, and ultimately preserves cellular structural integrity [26,27]. Conversely, high-temperature storage (25 °C) accelerates ROS-induced oxidative damage. This oxidative stress, coupled with intense microbial metabolism, leads to the hydrolysis of structural proteins and polysaccharides in *T. fuciformis*, resulting in a sharp decline in firmness and an abnormal increase in surface viscosity [28,29]. Concurrently, severe browning reactions (indicated by decreased L* and a* values, alongside increased b* values) occurred at higher temperatures. This color degradation is likely attributable to sustained polyphenol oxidase activity at elevated temperatures, as well as the accumulation of colored metabolic by-products resulting from extensive microbial contamination [30,31]. Ultimately, these microscopic physicochemical alterations manifest macroscopically as a rapid decline in overall sensory scores.

Accelerated Shelf-Life Testing (ASLT) utilizing the Arrhenius equation heavily relies on the selection of robust predictive indicators. In this study, although both TBC and MDA strongly correlated with product spoilage and adequately fit zero-order reaction kinetics (*R*^2^ > 0.95), model validation revealed a stark contrast in their predictive accuracy. The TBC-based Arrhenius model showed lower internal prediction error than the MDA-based model, suggesting that TBC may be a more suitable indicator for shelf-life estimation of this product within the tested temperature range. In contrast, the MDA-based model exhibited an unacceptable relative error of 70.67% at 25 °C. This discrepancy can be scientifically explained by the inherent differences between microbiological and chemical kinetics. As the direct cause of spoilage in high-moisture RTE foods, TBC generally follows strict thermodynamic temperature dependencies until the substrate is entirely depleted. However, MDA is a secondary intermediate product of lipid oxidation. At elevated temperatures (25 °C) and during the late stages of spoilage, MDA may further degrade or react with proteins under high thermal stress [32]. Consequently, MDA accumulation rates lose their stable linear relationship with temperature, causing the Arrhenius equation to significantly overestimate or underestimate its concentration. This finding suggests that for highly perishable, high-moisture edible fungus products, primary microbial indicators (TBC) are far more reliable for kinetic modeling than secondary chemical oxidation markers.

In summary, several key insights emerge from the comparative modeling approach employed in this study. First, for high-moisture RTE mushroom products, microbiological indicators (TBC) provide superior predictive performance compared to chemical oxidation markers (MDA) for Arrhenius-based shelf-life modeling, likely because microbial growth is the primary spoilage mechanism and follows a more predictable temperature dependence. Second, the selection of quality indicators for shelf-life modeling cannot rely solely on statistical correlation (both TBC and MDA showed high correlations with spoilage and adequate kinetic fits) but must also consider the mechanistic relationship between the indicator and the spoilage process, as well as validation performance. Third, zero-order kinetics adequately describe both microbial growth and lipid oxidation in this product within the tested storage period. Fourth, the Arrhenius model, while practically useful for guiding cold-chain management within the tested temperature range, has important limitations when built from only three temperature points, and its predictions should be treated as estimates rather than exact values, particularly for temperatures outside the tested range.

Several limitations of the current study should be acknowledged. First, the spoilage microorganisms were not identified to the species level; future studies should employ 16S rRNA sequencing or MALDI-TOF mass spectrometry for microbial community characterization. Second, pathogen-specific analysis was limited to bongkrekic acid; comprehensive testing for foodborne pathogens (coliforms, Escherichia coli, Staphylococcus aureus, Salmonella spp., and Listeria monocytogenes) is recommended in future work to fully characterize the safety profile of this product. Third, pH and water activity were measured only at the initial time point and should be monitored dynamically throughout storage in future studies to better understand their roles in spoilage dynamics. Fourth, the Arrhenius model was built using only three temperature points (*n* = 3), which limits the statistical power of the regression, and model validation was performed at the same temperatures used for model construction, representing internal rather than independent external validation. Future studies should include independent validation at additional temperatures (e.g., 10 °C or 20 °C) and under dynamic (fluctuating) temperature conditions simulating real cold-chain logistics. Fifth, while safety testing was performed before sensory evaluation of aged samples, the residual risk associated with tasting microbiologically aged products cannot be completely eliminated. Sixth, analytical method validation for bongkrekic acid and MDA, although improved in this revision, would benefit from inter-laboratory validation. Seventh, the use of Arrhenius-based modeling for microbial growth, while providing acceptable predictions in this study, may be less suitable for products exhibiting pronounced lag phases or nonlinear growth patterns; future work should compare predictions from the Arrhenius approach with those from dedicated predictive microbiology models (e.g., modified Gompertz, Baranyi, or square-root models). Despite these limitations, the present study provides a practical framework for shelf-life modeling of novel RTE mushroom products and identifies key areas for future investigation.

## 5. Conclusions

In this study, the quality deterioration kinetics of ready-to-eat *T*. *fuciformis* cold dishes under various storage temperatures were systematically evaluated to establish an Arrhenius-based shelf-life prediction model. The shelf-life endpoints, determined by interpolation to 5.0 log CFU/g, were 19.01, 5.53, and 1.58 days at 5 °C, 15 °C, and 25 °C, respectively. Throughout the storage period, elevated temperatures significantly accelerated product spoilage, characterized by increases in TBC, viscosity, yellowness (b*), and MDA content, alongside declines in firmness and overall sensory acceptability. The product reached its microbiological safety threshold well before any detectable bongkrekic acid accumulation, indicating that the current processing conditions inherently mitigate the risk of BGC toxin formation. Although both TBC and MDA kinetics followed zero-order reaction models, internal validation demonstrated that the TBC-based Arrhenius model showed lower internal prediction error than the MDA-based model within the tested temperature range. Conversely, the MDA-based model yielded larger deviations, suggesting that TBC is a more reliable biological indicator for the shelf-life prediction of this product. The TBC kinetic model provides a preliminary practical reference for cold-chain management, but further independent validation is required before industrial-scale application. However, the model should be further validated at independent temperatures and under dynamic cold-chain conditions before industrial-scale implementation.

## Figures and Tables

**Table 1:** Pearson correlation coefficients among various indices of RTE *T. fuciformis* cold dishes at three storage temperatures.

Temperature	Quality Parameter	Sensory Score	L*	a*	b*	Firmness	Viscosity	TBC	MDA
5 °C	Sensory score	1	0.060	−0.482	−0.966 **	0.994 **	−0.987 **	−0.991 **	−0.997 **
	L*		1	0.834 **	0.107	0.041	0.058	−0.092	−0.107
	a*			1	0.609 *	−0.492	0.585 *	0.460	0.440
	b*				1	−0.963 **	−0.987 **	0.964 **	0.947 **
	Firmness					1	−0.984 **	−0.991 **	−0.989 **
	Viscosity						1	−0.984 **	0.975 **
	TBC							1	0.987 **
	MDA								1
15 °C	Sensory score	1	−0.598	−0.551	−0.923 **	0.980 **	−0.974 **	−0.997 **	−0.998 **
	L*		1	0.783 *	0.791 *	−0.640	0.705	0.552	0.603
	a*			1	0.676	−0.589	0.078	0.504	0.553
	b*				1	−0.967 **	0.963 **	0.906 *	0.935 **
	Firmness					1	−0.977 **	−0.976 **	−0.987 **
	Viscosity						1	0.962	0.975 **
	TBC							1	0.994 **
	MDA								1
25 °C	Sensory score	1	−0.374	−0.883	−0.960 *	−0.985 *	−0.994 **	−0.979 *	−0.980 *
	L*		1	0.730	0.560	−0.213	0.292	0.181	0.226
	a*			1	0.974 *	−0.804	0.827	0.785	0.889
	b*				1	−0.915	0.922	0.904	0.889
	Firmness					1	−0.991 **	−0.999 **	−0.979
	Viscosity						1	0.987 *	0.996 **
	TBC							1	0.975 *
	MDA								1

Note: “*” means significant, *p* < 0.05; “**” means extremely significant, *p* < 0.01.

**Table 2:** Kinetic model parameters based on MDA and total bacterial count.

	Temperature	Zero-Order Reactions			First-Order Reaction		
		k	R^2^	∑R^2^	k	R^2^	∑R^2^
TBC	5 °C	0.1897	0.9886	2.9413	0.0606	0.9712	2.8960
	15 °C	0.8215	0.9590		0.2271	0.9925	
	25 °C	3.2959	0.9937		0.9700	0.9323	
MDA	5 °C	0.0207	0.9903	2.9328	0.0551	0.9929	2.9035
	15 °C	0.0714	0.9723		0.1970	0.9898	
	25 °C	0.1867	0.9702		0.8880	0.9208	

**Table 3:** Actual and predicted shelf life of RTE *T. fuciformis* cold dishes.

Quality Parameter	Temperature (°C)	Final Sampling Point (d)	Interpolated TBC-Based Shelf-Life Endpoint (d)	Relative Error (%)	Mean Relative Error (%)
TBC	5	22.00	19.01	13.59	8.92
	15	6.00	5.53	7.83	
	25	1.50	1.58	5.33	
MDA	5	22.00	19.53	11.22	30.41
	15	6.00	6.56	9.33	
	25	1.50	2.56	70.67	

## Data Availability

The original contributions presented in this study are included in the article/Supplementary Materials. Further inquiries can be directed to the corresponding author.

## Supplementary Materials

The following supporting information can be downloaded at: https://www.mdpi.com/article/10.3390/foods15132260/s1; Table S1: Sensory evaluation criteria for ready-to-eat *Tremella fuciformis* cold dishes. Figure S1: Dried and rehydrated *T. fuciformis*.
