# Supplementary material for: Quality Deterioration Kinetics and Arrhenius-Based Shelf-Life Prediction of Ready-to-Eat Tremella fuciformis Cold Dishes
Source: Foods. 2026 Jun 24;15(13):2260. doi: 10.3390/foods15132260 (PMC13361796; doi:10.3390/foods15132260)

Table S1. Sensory evaluation criteria for ready-to-eat *Tremella fuciformis* cold dishes.

Evaluation scale: Each attribute (color, aroma, texture, taste) is scored by 10 trained panelists. The overall sensory score is the sum of the four attribute scores (maximum 80). A total score ≥ 50 was defined as the minimum acceptable quality (sensory rejection threshold), corresponding to the overall sensory quality at the microbiological shelf-life endpoint (TBC = 5.0 log CFU/g).

Score	Color	Aroma	Texture	Taste
**16–20 (Excellent)**	Characteristic yellowish-white color of *T. fuciformis*; uniform and bright; no browning or discoloration.	Characteristic fresh mushroom aroma; no off-odor; clean and pleasant.	Firm and elastic; characteristic crunchy texture; smooth surface without excessive viscosity.	Characteristic umami taste of *T. fuciformis*; well-balanced seasoning; no off-flavor or rancidity.
**11–15 (Good)**	Slightly dull or pale; very minor browning at edges; color still acceptable.	Mild mushroom aroma; slight reduction in freshness; no obvious off-odor.	Slightly softened but still acceptable; minor increase in surface viscosity; elasticity partially retained.	Slightly reduced umami; seasoning still balanced; no perceptible off-flavor.
**6–10 (Fair)**	Noticeable browning or discoloration; dull appearance; color deterioration evident.	Weak mushroom aroma; slightly stale or fermented note detectable; borderline acceptability.	Obviously softened; noticeable surface viscosity; loss of crunchiness; texture deterioration apparent.	Markedly reduced umami; seasoning imbalance perceptible; slight off-flavor or staleness.
**1–5 (Poor/Rejected)**	Severe browning or darkening; color unacceptable; visible microbial colonies possible.	Strong off-odor; fermented, sour, or putrid smell; completely unacceptable.	Severely degraded texture; excessive viscosity or sliminess; complete loss of structural integrity.	Strong off-flavor; rancid, sour, or bitter taste; completely unacceptable for consumption.

Figure S1 Dried and rehydrated *T. fuciformis*

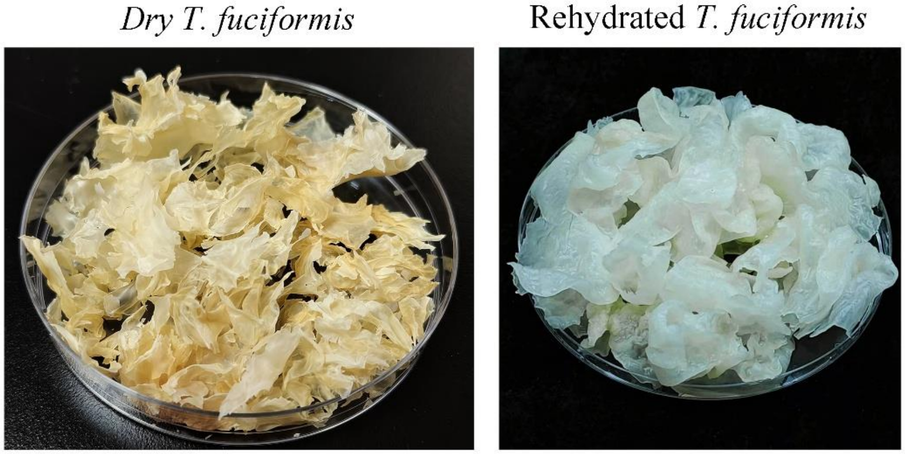

Supplement: Supplementary file 1 [file foods-15-02260-s001.zip › foods-4343096-supplementary.pdf]
